# Efficient carrier multiplication in CsPbI_3_ perovskite nanocrystals

**DOI:** 10.1038/s41467-018-06721-0

**Published:** 2018-10-10

**Authors:** Chris de Weerd, Leyre Gomez, Antonio Capretti, Delphine M. Lebrun, Eiichi Matsubara, Junhao Lin, Masaaki Ashida, Frank C. M. Spoor, Laurens D. A. Siebbeles, Arjan J. Houtepen, Kazutomo Suenaga, Yasufumi Fujiwara, Tom Gregorkiewicz

**Affiliations:** 1 Institute of Physics, University of Amsterdam, Science Park 904, 1098 XH Amsterdam, The Netherlands; 2 Division of Materials and Manufacturing Science, Graduate School of Engineering, Osaka University, 2-1 Yamadaoka, Suita, Osaka 565-0871 Japan; 3 Department of Physics, Osaka Dental University, 8-1 Kuzuha-Hanazono, Hirakata, Osaka 573-1121 Japan; 4 Graduate School of Engineering Science, Osaka University, 1-3 Machikaneyama, Toyonaka, Osaka 560-8531 Japan; 5 Department of Physics, Southern University of Science and Technology, Shenzhen, 518055 China; 6 Faculty of Applied Sciences, Delft University of Technology building 58, van der Maasweg 9, 2629 HZ Delft, The Netherlands; 7 National Institute of Advanced Industrial Science and Technology (AIST), AIST Central 5, Tsukuba, 305-8565 Japan

## Abstract

The all-inorganic perovskite nanocrystals are currently in the research spotlight owing to their physical stability and superior optical properties—these features make them interesting for optoelectronic and photovoltaic applications. Here, we report on the observation of highly efficient carrier multiplication in colloidal CsPbI_3_ nanocrystals prepared by a hot-injection method. The carrier multiplication process counteracts thermalization of hot carriers and as such provides the potential to increase the conversion efficiency of solar cells. We demonstrate that carrier multiplication commences at the threshold excitation energy near the energy conservation limit of twice the band gap, and has step-like characteristics with an extremely high quantum yield of up to 98%. Using ultrahigh temporal resolution, we show that carrier multiplication induces a longer build-up of the free carrier concentration, thus providing important insights into the physical mechanism responsible for this phenomenon. The evidence is obtained using three independent experimental approaches, and is conclusive.

## Abstract (web summary)

In semiconductor nanocrystals, efficient carrier multiplication counteracts hot carrier thermalization, increasing the overall carrier generation yield. Here, de Weerd et al. observe a quantum yield of up to 98% in CsPbI_3_ nanocrystals as a result of efficient carrier multiplication.

## Introduction

In the process of photoexcitation of a semiconductor, an electron in the conduction band and a hole in the valence band are created, forming an electron–hole (e–h) pair. The photo-generated e–h pair possesses typically an excess energy, equal to the difference between the band gap value of the material and the absorbed photon energy. The hot electron and hole may lose their excess energy by cooling to the band edge by phonon scattering. However, if the excess energy reaches a certain threshold, an interaction between a hot electron (hole) and other valence electrons (holes) can take place instead, such that a second e–h pair is generated. In bulk semiconductors, this phenomenon is known as impact ionization^1,2^ and was first observed in crystalline bulk semiconductors Si and Ge^3^. In the case of semiconductor nanocrystals (NCs), impact ionization is more often referred to as multiple exciton generation or carrier multiplication (CM), and its probability can be enhanced. CM is accompanied by Auger recombination (AR), which is the reverse process of impact ionization^4^: an e–h pair can recombine, giving up its energy to another electron or hole, thus increasing its excess energy and creating a hot carrier. Sequential CM and AR can continue until the hot e–h pair has cooled below the CM threshold, e.g., by phonon scattering.

In the last two decades, semiconductor NCs have been widely investigated for their size-tunable properties. As the NC size decreases and approaches the Bohr radius of the particular material, quantum confinement sets in. As such, the nanoparticle dimensions, and not the e–h Coulomb coupling strength, defines the exciton spatial confinement. Upon confinement, the wave functions of the electron and hole are modified and eventually discrete energy levels replace the continuous energy bands of the bulk material, while the band gap increases^5,6^. Because of the strong confinement, carrier–carrier Coulomb interactions are enhanced which can give priority to decay via AR, and reversibly, to efficient CM by hot carriers^7–9^. Employing CM for photovoltaic devices has already proved its benefit and usefulness^10^. In particular, a photovoltaic power conversion efficiency up to ~44% is expected for cells that make optimal use of CM^11,12^, surpassing the well-known Shockley–Queisser limit of ~33%^13^. Indeed, an external photocurrent quantum efficiency (the ratio of photocarriers collected by an external circuit to the number of incident photons) exceeding 100% has been reported^10^. Previously, CM has been demonstrated in many semiconductor (nano)structures, e.g., PbSe, PbS, CdSe, Si, Ge and graphene^10–12,14–30^, as reviewed by Smith and Binks^31^. Until now, however, CM has not been reported for perovskites. These materials are at the moment intensively researched for numerous applications. Perovskites attract considerable attention because of their outstanding optical and electrical properties, defect tolerance and low production costs^32–37^. Recently, the all-inorganic perovskite NCs (IP-NCs) are of interest, featuring extremely efficient emission^38,39^ and fast radiative recombination^40^. They combine the advantages of perovskites and NCs and, being free from the organic component, offer better stability than the more popular hybrid organic–inorganic perovskites^39^. Moreover, due to the recent demonstration of a stable solar cell based on CsPbI_3_ NCs^41^, this material has changed its status from being a scientific curiosity to a highly promising new alternative for perovskite-based applications.

In the case of IP-NCs, CM would be of great fundamental impact on this upcoming material and directly beneficial to its application in novel optoelectronic nanodevices, most notably photodetectors, while the band gaps of the currently available IP-NCs are still too large for practical impact in solar cells. Nevertheless, the research continues vigorously and rapid progress is being made—see, e.g., ref^42^ for a very recent report on the possibility of multiple exciton generation in CsPbBr_3_ upon nonlinear absorption at sub-band energies. That is why CM in IP-NCs is of interest and has been investigated further. Previous research conducted on IP-NCs NCs, failed to reveal CM: comparison of PL decay dynamics upon excitation with high (*hν* > 2*E*_gap_) and low (*hν* < 2*E*_gap_) energy photons did not show any signatures of CM^43^.

Here, we report on the observation of efficient CM in a colloidal dispersion of CsPbI_3_ NCs, with a band gap energy of 1.78 eV. We explicitly demonstrate the CM effect using ultrafast transient absorption (TA) spectroscopy in a variety of experimental approaches. By comparing carrier transients at different pump photon energies, we demonstrate the fingerprint of CM in the form of a fast component induced by AR of multiple e–h pairs appearing in the same NC. The observation is made both for the induced absorption and induced bleach. We confirm the CM effect and evaluate its efficiency by measuring the carrier generation rate as a function of excitation energy. We find an efficiency of 98 and 97% as extracted from the photo-induced bleach (PIB) and photo-induced absorption (PIA) dynamics respectively. In addition, we investigate in detail the dynamics for above threshold pumping, on a picosecond time scale. This reveals that the CM process coincides with a longer build-up of the free carrier concentration and provides new and important insights into the CM phenomenon. Moreover, we reproduce our investigations at an independent experimental setup, with somewhat different characteristics, and on newly synthesized materials. In that way, we provide an unambiguous evidence of CM in all-inorganic CsPbI_3_ NCs.

## Results

### Synthesis and microscopic characterization

**Fig. 1:**
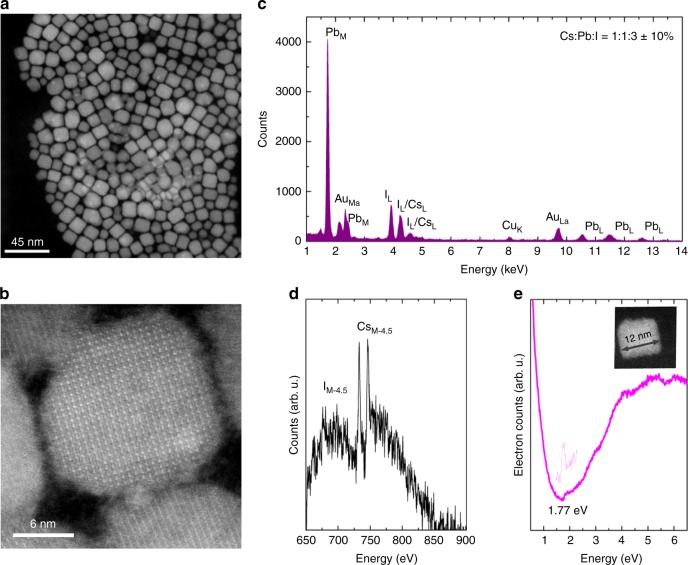
Microscopic characterization of the CsPbI_3_ nanocrystals. **a** Annular dark field image of the freshly drop casted sample. **b** Atomic resolution scanning transmission microscopy image of a nanocrystal showing clearly their mostly cubic structure. **c**, **d** Energy dispersive X-ray spectrum of the same sample (**c**) and the core-loss electron energy loss spectrum (**d**) where mainly Cs, Pb and I are detected. The Cs:Pb:I ratio is 1:1:3 ± 10%, as determined from the quantification of the energy dispersive X-ray signal for the L-lines of the corresponding elements, which suggests (mostly) the perovskite composition. **e** Low-loss electron energy loss spectrum of a large, 12 nm nanocrystal, with a band gap energy of 1.77 eV. The dotted line indicates the first derivative of the spectrum around the absorption onset, where its maximum is taken as the NC band gap energy

The CsPbI_3_ NCs were synthesized by a wet-chemistry method, following the slightly modified protocol described in refs^39^ and ^41^. We used a synthesis temperature of 180 °C in order to steer the production towards NCs with a small band gap energy, i.e., larger size. Moreover, large NCs have a larger absorption cross section, and therefore are excited preferentially. We measured a photoluminescence quantum yield (PL QY) of 42.4 ± 7 % at excitation of energy of 3.1 eV (400 nm, see Supplementary Fig. 1 for the excitation dependent PL QY), which is similar to previously reported values^39,41^, and is expected for NCs with a large size (the Bohr radius is ~6 nm for this material therefore the NCs are in the weak confinement regime). The structural characteristics of the NCs were determined using a state-of-the-art low-voltage monochromatic scanning transmission electron microscope (STEM) with a spatial resolution below 1.6 Å. Figure 1a shows the annular dark field STEM image of the drop-casted NCs with an average size of 11.5 ± 0.6 nm (see also Supplementary Fig. 2 and detailed information in the Supplementary Information on materials and methods). A high-resolution annular dark field image of a 12 nm NC is shown in Fig. 1b. Here, the (nearly) cubic arrangement of the atoms is clearly observed, consistent with the perovskite structure. This is further analyzed by simultaneously performing energy dispersive X-ray spectroscopy (Fig. 1c) for elemental identification and core-loss electron energy loss spectroscopy (EELS, Fig. 1d). Mainly Cs, Pb and I are detected and the Au signal arises from the reflection of the TEM grid. The Cs:Pb:I ratio, determined from the quantification of the energy dispersive X-ray signal under the L-lines of the corresponding elements, is 1:1:3 ± 10%, confirming that (mostly) NCs of CsPbI_3_ perovskite have been formed. At high losses, two distinctive peaks at 733 eV and 746 eV are observed, corresponding to Cs_M4.5_, and the broader peak at 700 eV which corresponds to the delayed feature of the I_M4.5_ ionization edge. To determine the band gap of a single NC, imaging and valence-loss EELS collections were performed in parallel, with a high energy resolution (zero loss peak of ~50 meV)^44–46^. In this way, the structural parameters of a single NC were determined, while simultaneously, its low-loss EEL spectrum was recorded. A characteristic step appears in the low-loss EEL spectrum, as an electron is excited from the top of the valence band to the conduction band, followed by a steady increase of the signal. This corresponds to the band-to-band absorption, whose magnitude grows as the density of states increases with energy, in analogy with optical absorption^47^. Fig. 1e shows the low-loss EEL spectrum with the characteristic step, and the simultaneously obtained annular dark field image of a 12 nm NC—which somewhat appears at the edge of the size distribution (see also Supplementary Fig. 2) and has specifically been chosen because CM is expected to commence for large NCs first. A band gap energy of 1.77 eV is determined by taking the maximum of the first derivative of the spectrum around the absorption onset, as shown by the dotted line.

### Optical characterization

**Fig. 2:**
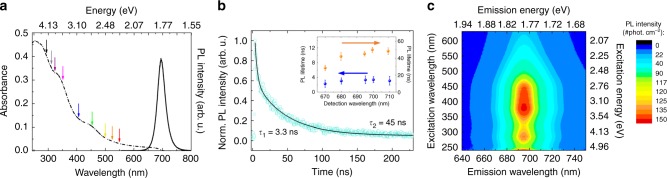
Optical characterization. **a** Absorbance (dotted) and PL (solid) spectra. The colored arrows indicate the photon energies used in the transient absorption experiment. **b** Time-resolved photoluminescence (PL) measurement for *λ*_det_ = 695 nm which was fitted using a bi-exponential function and yielding decay times *τ*_1_ = 3.3 ns and *τ*_2_ = 45 ns. The inset shows the obtained lifetimes for all detection wavelengths. The PL lifetimes with corresponding amplitudes are shown in the inset. The uncertainty is determined by the statistical error from the fitting method. **c** 2D contour plot of the PL excitation, showing the effect of excitation energy on the PL emission and intensity. A PL maximum is observed around <250 nm (>4.96 eV), which could be the first sign of carrier multiplication. The excitation and emission intensities are corrected for the wavelength dependent components of the setup and spectral sensitivity

Fig. 2a shows the ensemble optical absorption of the NCs (dotted line) and the PL spectrum (solid line) with its maximum at 1.78 eV (695 nm). The colored arrows indicate the excitation photon energies that were employed in the TA experiments. The PL lifetimes were measured using a picosecond pulsed diode laser (*λ*_exc_ = 375 nm) as an excitation source. The normalized decay dynamics for *λ*_det_ = 695 nm, and extracted lifetimes for the remaining detection wavelengths are shown in Fig. 2b. The transients could be fitted with a bi-exponential function, yielding PL lifetimes of 3.3 and 45 ns with a respective amplitude ratio of 1:2, in a good agreement with previous reports^41,43,48^. Fig. 2c shows the PL excitation data where distinctive maxima are observed for excitation energies around 2.8–3.3 eV (440-375 nm), 4.3 eV (290 nm), and 4.96 eV (250 nm). Similar features have been observed before and the maxima at lower energies were attributed to higher energy states observed in the absorption spectrum^48^. In that specific case, the PL intensity was significantly reduced for higher excitation energies (>4.6 eV/<270 nm), which was explained by the loss of excess energy through non-radiative recombination. This is clearly not the case in this study: above 4.96 eV (250 nm), we observe a high-intensity emission maximum. We note that, in our experiment, the obtained emission and excitation spectra are corrected for possible wavelength dependent characteristics of the experimental setup. The data are collected as the number of emitted photons per unit area (which remains unchanged throughout the experiment), and in that regard, resembles a PL QY determination. In addition, we recall that in the past for above threshold excitation in Si NCs, an increase of the PL QY was observed which was assigned to CM^20^. As such, the observed increase of the PL excitation above 4.96 eV, taken together with the NCs absorption spectrum (Fig. 2a), suggests an increase in PL QY appearing due to CM^20^. Unfortunately, an observation of this phenomenon at still higher excitation energies was not possible due to limitations of the setup.

### Transient absorption spectroscopy

The peculiarity of the PL excitation spectrum in Fig. 2c motivated the use of TA spectroscopy, which in the past has proved very successful in CM investigation in nanostructures^15,16,21,49^. For direct band gap semiconductors, the interband PIB is often used to study the CM process, while for indirect band gap semiconductors this is typically weak, therefore it is easier to monitor the PIA, caused by intra-band transitions of (free) carriers generated by the pump pulse. First, a strong pump pulse provides band-to-band excitation, which in NCs leads to generation of e–h pairs. After photoexcitation, these initially hot e–h pairs relax and bleach the optical absorption at the band edge. A second, weaker pulse probes the carrier concentration as a function of the pump–probe delay time. As a result, the transmittance for photons with energy near the (direct) band gap increases, and yields a negative PIB signal. The TA signal is defined as the difference of the absorbance or optical density (OD) with and without pump and is obtained as

1 $$\Delta {\mathrm{OD}}({\mathrm{t}},\lambda ) = {\mathrm{log}}_{10}\frac{{{{I}}_{{\mathrm{probe}}}(\lambda )}}{{{{I}}_{{\mathrm{pump}} + {\mathrm{probe}}}(\lambda )}},$$

where *I*_probe_(*λ*) and *I*_pump+probe_ (*λ*)′ are the transmitted probe fluences with the pump pulse off and on, respectively. Two approaches are generally considered: firstly the *A*/*B* method applied by Schaller et al. relying on the appearance of a fast component in the transient as the pump energy exceeds the CM threshold^50^, and secondly the excitation energy dependence of the number of generated carriers (i.e., carrier generation yield), determined by the PIB or PIA amplitude as a function of the absorbed photon fluence^15,16,51,52^. Here, we apply both methods, yielding consistent results. We also consider both the PIB as well as PIA dynamics, whose results are in agreement with each other. In addition, since ultrafast spectroscopy setups often have their own characteristics and elements, we repeated our investigations at two different experimental stations (see the Methods sectionfor the details of the setups used to obtain the presented data). Our approach (using multiple methods to identify CM and repeating the experiments at two stations) is unique, and was followed because of the great experimental difficulty of (ultrafast) CM experiments, which are prone to artefacts, as evidenced by the existing literature see, e.g., ref^15^.

### The fingerprint of CM

**Fig. 3:**
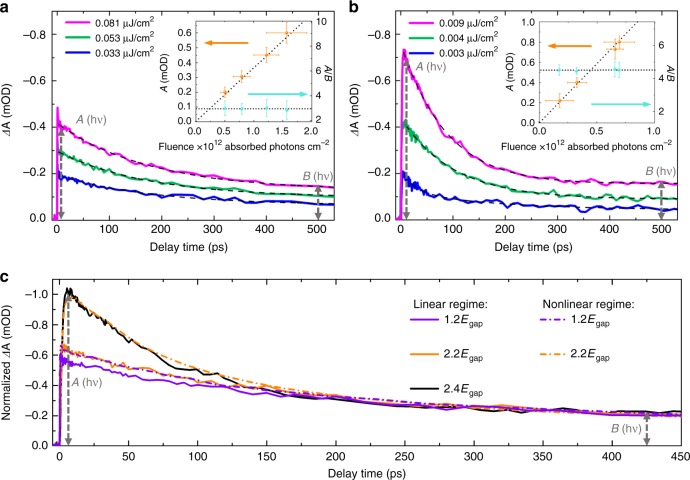
Transient absorption dynamics. **a**, **b** Dynamics below (**a**) and above (**b**) the carrier multiplication (CM) threshold, i.e., at pump wavelengths of 500 nm (2.48 eV) and 295 nm (4.2 eV), respectively. The dashed lines represent the exponential fit to the data. The appearance of the additional fast component when pumping at 4.2 eV is the fingerprint of CM. The insets shows the initial transient amplitude *A* and its ratio to the single exciton decay tail *A*/*B*, as a function of the absorbed photon fluence, demonstrating the single photon absorption (linear) regime. All dynamics are measured at probe wavelengths around the photo-induced bleach maximum (680 nm) by integrating the signal from 675 to 685 nm. The latter determines the error bars in the *y*-direction. The error in the *x*-direction arises from small fluctuations in the pump power. **c** Linear vs. nonlinear regime, showing the decay through Auger recombination with pumping outside the linear regime (i.e., by multi-photon absorption) and through CM, yields the same dynamics

Fig. 3 shows the transient PIB measured around its maximum at *λ*_probe = _680 nm (obtained by integrating the signal between 675 and 685 nm). Here, the results for two excitation wavelengths are compared: below and above the CM threshold, at *λ*_exc = _500 nm (1.4*E*_gap_, Fig. 3a) and *λ*_exc = _295 nm (2.4*E*_gap_, Fig. 3b), respectively (see Supplementary Fig. 3 for all transient PIB dynamics measured in this study). While performing our TA measurements, we have maintained the number of absorbed photons per NC at a very low level (〈*N*_exc_〉« 1) within the so-called linear regime. This is confirmed by the fact that the initial amplitude *A* of the TA dynamics grows linearly with the absorbed photon fluence (orange dots in the insets of Fig. 3a, b), and particularly by the ratio between the initial amplitude *A* and the tail amplitude *B* (*A*/*B*) which remains invariant within the investigated absorbed photon fluence range (turquoise dots). Specifically, from the latter observation, multi-photon absorption can be completely excluded. As such, the observed fluence increase in this range yields only a higher number of excited NCs while maintaining 〈*N*_exc_〉« 1: under these excitation conditions each NC contains at most 1 e–h pair, following absorption of a single photon.

Considering both excitation energies, we note that the behavior of the transient PIB signal is remarkably different: in both cases the dynamics show a tail with a relaxation time which fits the displayed time window (500 ps). However, for the UV excitation, an additional fast component appears. This is commonly taken as a fingerprint of CM, which we will now briefly explain. Here, we follow the reasoning as originally proposed by Schaller et al.^50^. which has been employed in many studies of CM before^15,21^—and see also ref^27^. A hot carrier can induce CM, if its excess energy is greater than the threshold energy, which must at least be equal to the NC band gap. A single photo-generated hot e–h pair loses its excess energy by generating an additional e–h pair. This higher exciton multiplicity causes the amplitude increase of the PIB/PIA signal at the short time scale. Subsequently, the multiple excitons localized within the same NC decay through AR. This process typically occurs within 10-100 ps and gives rise to the initial fast component in the measured dynamics^53^. The experimental data shown in Fig. 3 could be fitted with a double and triple exponential function for below and above CM threshold pumping respectively. This yields *τ*_1_ = ~ 89 ps for the fast component for above threshold pumping which agrees with previously reported time constants for the AR process^43,53,54^. After the multi-exciton relaxation process, a single exciton remains, which decays through non-radiative and radiative recombination processes, depending on the characteristics of individual NCs, (because the ensemble PL QY < 100%) giving rise to the slow decay. The experimentally found slow decay time constants are *τ*_2_ = ~190-200 ps and *τ*_3 = _~3300 ps (see also Supplementary Fig. 4), where the latter agrees with the radiative lifetime determined from the TR-PL measurements.

To prove that the initial fast component is indeed due to AR, we show that it can be reproduced by increasing the pump fluence at below-threshold pump photon energy, such that multi-photon absorption occurs. Figure 3c shows the transients for the highest pump fluences in the linear regime (solid lines) which can be reached, at far below (1.2*E*_gap_, purple), just above (2.2*E*_gap_, orange), and above (2.4*E*_gap_, black) CM threshold excitation. The dynamics are normalized to the same *B* value such that the increase of the fast component can clearly be distinguished. Then, the pump fluence for the below-threshold excitation at 1.2*E*_gap_ is increased, until its initial amplitude *A* matches that of the transient obtained for the above-threshold excitation at 2.2*E*_gap_ in the linear regime. And similarly, the pump fluence at 2.2*E*_gap_ is increased until it reaches *A* for exciting at 2.4*E*_gap_ in the linear regime (dotted lines). It can clearly be seen that the transients obtained under these two excitation conditions (in- and outside the linear regime) are practically identical, which confirms that the initial fast component is induced by AR: the decay through AR of multiple e–h pairs induced by multi-photon absorption and by CM, has identical characteristics, as was anticipated.

### CM efficiency

**Fig. 4:**
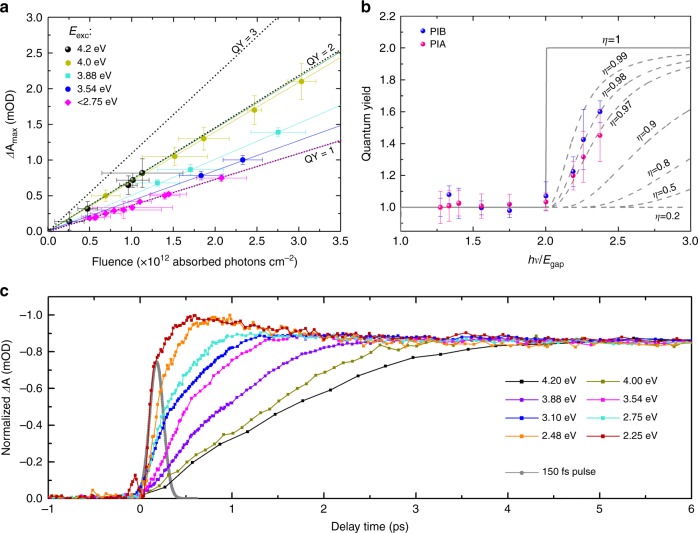
Carrier multiplication efficiency and Auger recombination. **a** *Δ*A as a function of the absorbed photon fluence. The solid lines represent a linear fit through the data points. **b** CM efficiency plotted as a function of excitation energy normalized to the band gap energy of the CsPbI_3_ nanocrystals. The blue and pink data points correspond to the yield calculated from the *A*/*B* ratios deduced from the photo-induced-bleach and -absorption respectively. **c** Initial rise of the photo-induced bleach signal (normalized) which becomes slower when the nanocrystals are excited with higher photon energies, and carrier multiplication sets in. The error bars are determined by small fluctuations in the pump power (*x*-direction) and from integrating the dynamics between 4 and 8 ps yielding an upper and lower limit for *Δ*A (*y*-direction)

Having obtained the fingerprint of CM, we now study its dependence on the pump fluence and excitation energy. The initial amplitude *A* serves as a measure for the number of generated e–h pairs and is therefore directly associated with the CM efficiency. Following the approach of Beard et al.^55^, *Δ*A is related to the CM QY via:

2 $$\Delta {\mathrm{A}} = {\mathrm{QY}}F_{{\mathrm{abs}}},$$

where *F*_abs_ is the absorbed photon fluence. For each excitation wavelength and corresponding pump power, *F*_abs_ is calculated via

3 $$F_{{\mathrm{abs}}} = \frac{{{{P}}_{{\mathrm{pump}}}\lambda {\mathrm{OD}}(\lambda _{{\mathrm{pump}}})}}{{{f}}},$$

where *P*_pump_ is the pump power at the sample position and corrected for a possible mismatch in pump/probe overlapping area; *λ* is the excitation wavelength; OD(*λ*_pump_) is the optical density determined from the linear absorption measurement and *f* is the laser frequency, which is setup specifically. As has been demonstrated in Fig. 3a and b, *Δ*A has a linear function passing through the origin and it follows from equation (2) that its slope determines the carrier generation yield. Accordingly, Fig. 4a shows *Δ*A as a function of *F*_abs_. At each pump energy, this value is determined for all pump fluences in the linear regime (as previously explained, the linear regime is mainly confirmed through invariance of the *A*/*B* ratio of the decay transients). Without multiple carrier generation, i.e., below the CM threshold, this slope should remain constant for different pump energies, which is indeed the case. In Fig. 4a the pink data points correspond to the pump energies *E*_exc_ < 2.75 eV < 2*E*_gap_ and follow the same linearity, which subsequently determines the QY = 1 line. From this it follows that QY = 2 is determined by a doubling of *Δ*A at equal fluence. In that way, the slope of the linear function through *Δ*A for each specific pump energy is a measure for the QY efficiency. Note that in Fig. 4a the slope gradually increases for pump energies above the CM threshold, i.e., *E*_exc_ ≥ 3.54 eV ≥ 2*E*_gap_, rather than jumping to the QY = 2 position (blue and turquoise curves). This is among others a result of the NC size distribution on CM, which commences first for the large NCs, with a smaller band gap energy. We find that at 4.0–4.2 eV excitation (2.2–2.4 *E*_gap_) the *Δ*A lines approach QY = 2 (dark yellow and black). The error bars in the y-direction are determined by the averaging between *t* = 4–8 ps after photo-excitation, to extract *Δ*A. In the x-direction, the error is determined by the upper and lower values of the measured pump power in between consecutive measurements.

*Δ*A in Fig. 4a only takes into account the initial amplitude *A* as a measure for the number of e–h pairs. However, to determine the exact QY value, their multiplicity should be normalized to the *B* value since our transients do not decay to zero within the available time window. In that way, the experimental CM yield *φ* (*hν*) can be determined from the *A*/*B* ratio at a specific energy *E*_exc_ > 2*E*_gap_, scaled to the value corresponding to below CM threshold pumping, via

4 $$\varphi (h\nu ) = \frac{{A\left( {h\nu } \right)/B(h\nu )}}{{A\left( {1.2E_{{\mathrm{gap}}}} \right)/B(1.2E_{{\mathrm{gap}}})}}.$$

Figure 4b shows *φ*(*hν*) as a function of the pump energy normalized to the NCs band gap energy of 1.78 eV. In that way, an increase of the QY can be easily monitored and can start from 2, which is indeed what we observe. Here, a direct determination of the CM efficiency (*η*_CM_) can be made, following the model proposed in ref.^55^:

5 $${\mathrm{CM QY}} = \left( {\frac{{h\nu }}{{E_{{\mathrm{gap}}}}} - 1} \right)\eta _{{\mathrm{CM}}},$$

where *hν* is the incident pump photon energy. The characteristic step-like feature with its threshold energy at 2*E*_gap_ is obtained for *η*_CM_ = 1. Subsequently, as the conversion efficiency decreases, the sharp onset broadens as is indicated by the dotted lines. Here, we obtain a high CM conversion efficiency *η*_CM_ of ~98% and ~97%, as deduced from the dynamics around the PIB as well as the PIA maxima (see also Supplementary Figs. 5 and 6 for PIA dynamics and TA spectra respectively).

### Photo-bleach rise time

Now we consider the rise time of the PIB transients as a function of the excitation energy, which evidences the build-up of the free carrier population (Fig. 4c). Here, an abrupt increase appears when the CM threshold identified from our above discussed measurements, is reached—i.e., *E*_exc_ ≥ 2*E*_gap_. This delay in rise time is clearly longer than the experimental temporal resolution of the setup (see also Supplementary Fig. 7). It can be observed for the PIB as well as the PIA (see Supplementary Fig. 8), on a similar time scale. Its characteristic time constant is estimated as 1–3 ps, depending on the pump energy, in agreement with ref^49^. One could argue that the increase of the PIB signal build-up at higher excitation energies, could alternatively be explained by the larger excess energy of the photoexcited carriers and, consequently, the longer relaxation time necessary to reach the band edge states probed by the PIB^56^. However, such a possibility is at variance with firstly the observation that the effect is also visible for PIA, which probes all the carriers in the band, and secondly the persistence of the rapid increase of the signal rise, for excitation above the CM threshold. We therefore—tentatively—attribute this additional e–h pair generation time to the CM process.

## Discussion

The presented experimental results provide new and unique insights into the physical mechanism of the CM process. While CM has been investigated and modeled theoretically for some time now, no generally accepted model of its physical mechanism exists. In particular, very little is known on the material parameters governing the efficiency and the threshold of CM—the most important features determining the possible impact of CM for practical applications. The current observation provides here two important clues. First: the CM process is not instantaneous, the additional carriers appear clearly after those generated primarily upon photon absorption. And second: CM seemingly does not affect the initial increase of the PIB signal (the first 150 fs), which, depending on the pump energy is determined by a combination of carrier generation upon photon absorption and their cooling to the probed state^56^. The latter observation is especially insightful: since the experimentally observed cooling time is determined only by the relaxation between the lowest states in bands, this implies that the hot carrier losing its energy by CM is not directly transferred to the lowest state, and that the secondary carrier, created by CM, arrives in a state with a considerably longer relaxation time. We recall that such states (in the form of self-trapped excitons) have been investigated in the past for Si NCs^57–59^ and have recently been invoked also for IP-NCs^60^. We have also observed that the time constant of this very characteristic prolonged build-up of PIB signal depends on the NC size, shortening for smaller NCs—see Supplementary Fig. 9. Such a dependence has indeed been postulated by some theoretical models of CM^61^. Moreover, we have shown that the enhancement of the rise time is uniquely related to the onset of CM, and does not appear when multiple electron–hole pairs are generated by high-power subthreshold pumping—see Supplementary Fig. 10.

Finally we briefly address the possible origin of the different outcome of this study in comparison with the previous investigation^43^. In our opinion, this could be related to small differences in the material preparation. The synthesis of IP-NCs has been reported only in 2015^39^ and while the protocol is not very complicated, a quick literature scan readily reveals that properties of materials prepared by different groups are not identical. In the present case, one specific difference could be the local stoichiometry, especially close to the surface, and a possible formation of insulating inclusions, featuring strong absorption bands in the 3.5–4 eV range—this in analogy to the recently identified CsPbBr_3_/Cs_4_PbBr_6_ hybrids^62^. One could speculate that strong absorption at energies close to twice the band gap could promote CM^13^. One other important difference of the present study is the application of PIA/PIB rather than ultrafast PL spectroscopy, as used in ref^43^. It cannot be excluded that both techniques could probe different populations of carriers, with PL reflecting exclusively a population of the emitting state. In conclusion, we demonstrate efficient CM in CsPbI_3_ NCs from the transient PIB and PIA dynamics in TA spectroscopy, using a variety of experimental strategies. The occurrence of CM is identified through the observation of an additional fast component in the transient dynamics, recorded for above-threshold pumping, maintaining the experimental conditions 〈*N*_exc_〉 « 1. CM is further confirmed from the photo-excitation energy dependence of carrier generation yield as a function of pump fluence. CM commences just after the energy conserving threshold of *E*_exc_ ≥ 2*E*_gap_ of the large NC fraction (*d*_NC_ ~ 12 nm with *E*_gap_ = 1.77 eV) and features a CM QY up to 98%. In addition, coincident with the CM process a longer picosecond build-up of the free carrier concentration for high-energy pumping is observed.

## Methods

### Materials

The CsPbI_3_ NCs were synthesized following the protocol first reported by Protesescu et al. in 2015, applying a slight alteration^39^. To prepare the Cs-oleate, 0.814 g of Cs_2_CO_3_ is mixed with 40 mL of ODE and 2.5 mL of OA. The mixture is subsequently stirred at 150 °C in an inert atmosphere until the reaction is complete. Here, the reactants are dried for 1 h at 120 °C. To induce the formation of NCs, 5 mL of ODE and 0.188 mmol of PbI_2_ are dried in a N_2_ atmosphere for 1 h at 120 °C. After water removal, 0.5 mL of dried OA and 0.5 mL of dried OLA are added to the reaction flask, increasing the temperature to 180 °C. After the solvation of the PbI_2_ is complete, 0.4 mL of the Cs-oleate solution is injected (which is warmed up previously). The reaction takes a few seconds where after the NCs solution is cooled down quickly using an ice bath. The final product is purified using several centrifugation steps and is redispersed in hexane. The sample is diluted to reach the appropriate optical density («1 OD) for the spectroscopy experiments, and transferred to a quartz cuvette (UV grade).

### Experimental setups

In the TA experiment for UV pump energies (station 1, >4 eV), a Mai Tai-SP (Mountain View, U.S.A.) Diode-Pumped, Mode-Locked Ti:sapphire Laser, operating at 1 kHz with~100 fs pulse width and ~3mJ pulse energy for an output wavelength of 800 nm, is used as the coherent light source. A frequency regulator reduces the output frequency to 200 Hz. A beam splitter is used to separate the beam to generate the pump and probe pulses. An Optical Parametric Amplifier, TOPAS-C (Light Conversion, Vilnius, Lithuania) in combination with a BBO crystal, generates the desired pump energy. The pump beam is subsequently guided through a delay stage (SGSP 26-200). The probe signal consists of white light which is generated using a sapphire crystal, and is spectrally resolved before reaching the detector: a 0.5 m Imaging Triple Grating Spectrogram, SpectraPro 2500i (Acton Research Corporation, Acton, U.S.A.) and an air-cooled CCD camera, PIXIS 256 (Princeton Instruments, Trenton, U.S.A.) were used. For the remaining pump energies (station 2, <4 eV), the sample is excited with a ∼150 fs laser pulse (Light Conversion Pharos-SP operating at 2.5 kHz, combined with an Orpheus OPA) and probed using a multichannel detection of visible/near-infrared (500–800 nm) probe pulses (Ultrafast Systems Helios). The broad band (white light) probe pulses are generated by a sapphire crystal using the 1030 nm pump light. We note that the experiments at ‘low’ pump energies have been repeated at station 1 and subsequently compared. Those experiments yield equal dynamics and as such, validates the results (see Supplementary Fig. 11). The TA signal is defined as the difference of the optical density (OD) of the excited state and the ground state and is obtained as

6 $${\mathrm{OD}} = {\mathrm{log}}_{10}\left( {\frac{{{{I_{{\mathrm{total}}\,{\mathrm{incident}}\,{\mathrm{light}}}}}}}{{{{I_{{\mathrm{transmitted}}\,{\mathrm{light}}}}}}}} \right)$$

7 $$= \Delta {\mathrm{OD}} = {\mathrm{OD}}_{{\mathrm{pump}} + {\mathrm{probe}}} - {\mathrm{OD}}_{{\mathrm{probe}}}$$

8 $$= {\mathrm{log}}_{10}\left( {\frac{{{{I_{\mathrm{total}}}}}}{{I_{{\mathrm{pump}} + {\mathrm{probe}}}}}} \right) - {\mathrm{log}}_{10}\left( {\frac{{{{I_{\mathrm{total}}}}}}{{{{I_{\mathrm{probe}}}}}}} \right)$$

9 $$= {\mathrm{log}}_{10}\left( {\frac{{{{I_{{\mathrm{total}}}\times I_{{\mathrm{probe}}}}}}}{{I_{{\mathrm{pump}} + {\mathrm{probe}}}\times I_{\mathrm{total}}}}} \right)$$

10 $$= {\mathrm{log}}_{10}\frac{{{{I_{{\mathrm{probe}}}}}}}{{I_{{\mathrm{pump}} + {\mathrm{probe}}}}},$$

i.e.,

11 $$\Delta {\mathrm{OD}}(t,\lambda ) = {\mathrm{log}}_{10}\frac{{I_{{\mathrm{off}}}(\lambda )}}{{I_{{\mathrm{on}}}(\lambda )}},$$

where *I*_off_(*λ*) and *I*_on_(*λ*) are the transmitted probe fluences with the pump pulse off or on, respectively. While performing the TA experiments, the colloidal sample could be stirred to avoid potential charging, although, the stirring of the sample did not induce any difference in the measured dynamics.

A LAMBDA 950 UV/VIS/NIR spectrophotometer, PerkinElmer, is used to measure the absorbance/optical density with has an excitation range of *E*_det_ = 0.4–5.6 eV. Here, the absorption spectrum of the solvent (hexane) is measured separately and subtracted from the CsPbI_3_ NCs spectrum.

A Jobin Yvon FluoroLog spectrofluorometer, Horiba, is used to measure the PL excitation. As an excitation source, a 450 W xenon lamp (250–700 nm) is equipped providing a range of excitation wavelengths being coupled to a monochromator. The emission from the sample is always collected in a right-angle geometry. All spectra are corrected for the spectral sensitivity of the spectrofluorometer .

The PL QY is measured by placing the sample in an integrating sphere. Here a 150 W xenon lamp coupled to a spectrometer (Solar, MSA-130) is used as an excitation source, providing a selection of excitation wavelengths. The excitation and emission light is scattered diffusively in the integrating sphere. This is measured both for the cuvette containing the CsPbI_3_ NCs and for the solvent. The respective emission and excitation spectra are subtracted to calculate the QY. The spectra are recorded by a CCD (Hamamatsu).

The time-resolved PL measurements are performed using a LifeSpec II time-correlated single photon counting (TCSPC) spectrometer (Edinburgh Instruments). It has a 230-850 nm detection range (MCP-PMT). A diode laser with *λ*_exc_ = 375 nm (EPL series) provides a 100 ps pulse. A right angle between the excitation and emission beam paths is maintained to avoid detecting scattered excitation light.

### EELS and STEM

Prior to investigation, the NCs are freshly drop-casted onto a holey Au quantifoil TEM grid which is covered by a monolayer of graphene. As such, only NCs on top of graphene are measured. The substrate containing the NCs is left to dry to evaporate the solvent. The EELS experiments and simultaneous STEM imaging are performed using a JEOL ARM200 microscope with a Schottky thermal emission gun, operating at 15–60 keV. It is equipped with a probe delta corrector, a JEOL double Wien filter monochromator and a Gatan Quantum GIF spectrometer. All the experiments are performed in high-energy resolution mode using a slit of 0.5 µm. The probe beam has a zero loss peak with a FWHM around 50 meV, a ~8 pA current and a convergence semi-angle of 33 mrad. Switching to EELS collection mode, the semi-angle changes to 11 mrad. The sample is cooled down to −110 °C using liquid nitrogen, and a Gatan cryo-holder is used. This procedure reduces carbon redeposition as well as thermal excitation during imaging and EELS collection. In order to align the simultaneously high-loss spectrum, dual EELS acquisition is applied. This is done to correct for potential energy drift while performing the experiments. The size of the electron probe beam is 1.6 Å, when performing the low-loss EELS experiments^44–46^. All recorded data are raw, without any filtering.

## Electronic supplementary material

Supplementary Information

## Data Availability

All relevant data generated or analyzed during the current study are available from the corresponding authors on reasonable request.
