# Supplementary material for: Efficient carrier multiplication in CsPbI3 perovskite nanocrystals
Source: Nat Commun. 2018 Oct 10;9:4199. doi: 10.1038/s41467-018-06721-0 (PMC6180104; doi:10.1038/s41467-018-06721-0)
Supplement: Supplementary file 1 — Supplementary Information [file 41467_2018_6721_MOESM1_ESM.pdf]

SUPPLEMENTARY INFORMATION FOR:

Efficient Carrier Multiplication in $CsPbI_3$ Perovskite Nanocrystals

de Weerd & Gomez *et al.*

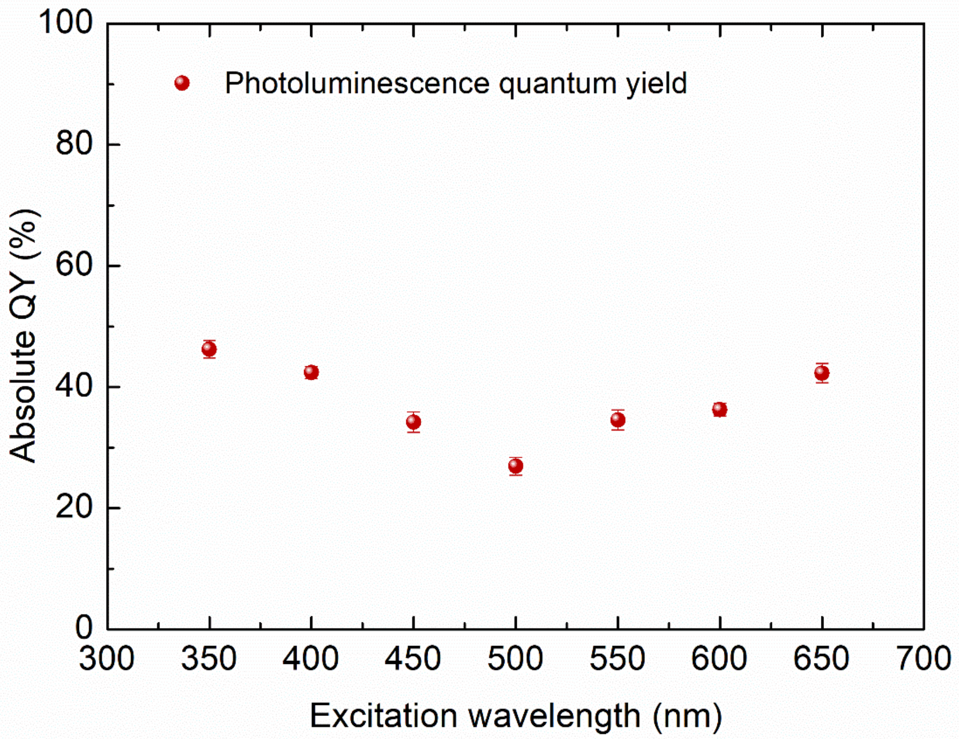

Supplementary Fig. 1: Absolute photoluminescence quantum yield. Using the integrating sphere method, the photoluminescence quantum yield has of the $CsPbI_3$ nanocrystals been determined as function of the excitation energy. We find an average value of ~40% which is expected for $CsPbI_3$ nanocrystals with a larger size. Error bars are determined from the statistical error from the fitting method and the excitation source fluctuations.

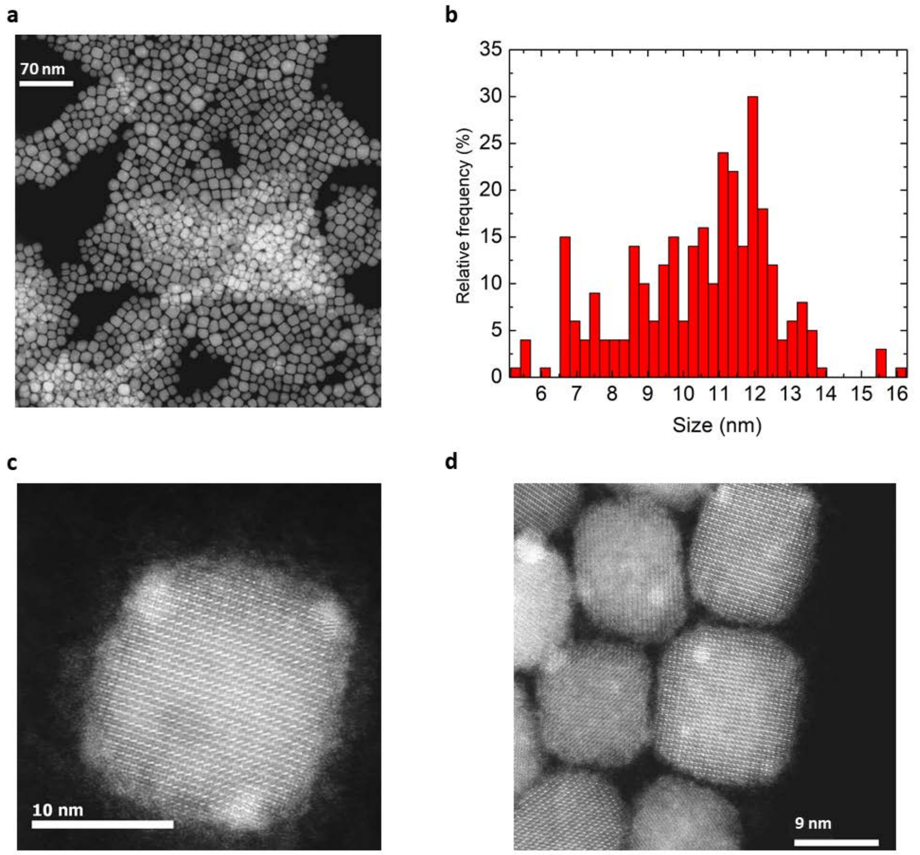

Supplementary Fig. 2: Annular dark field images of the $CsPbI_3$ nanocrystals and size distribution. (a) A larger area of the transmission electron microscope grid containing a freshly drop casted sample of the $CsPbI_3$ nanocrystals, already indicating that the average size of the nanocrystals is quite large. (b) The corresponding histogram to determine the average nanocrystals size (11.5 ± 0.6 nm). (c) Atomic resolution image of a single ~11 nm $CsPbI_3$ nanocrystal. (d) Atomic resolution annular dark field image of a nanocrystal cluster, showing that while some nanocrystals appear to have rounded corners, the cubic orientation of the atoms is preserved maintaining the perovskite structure.

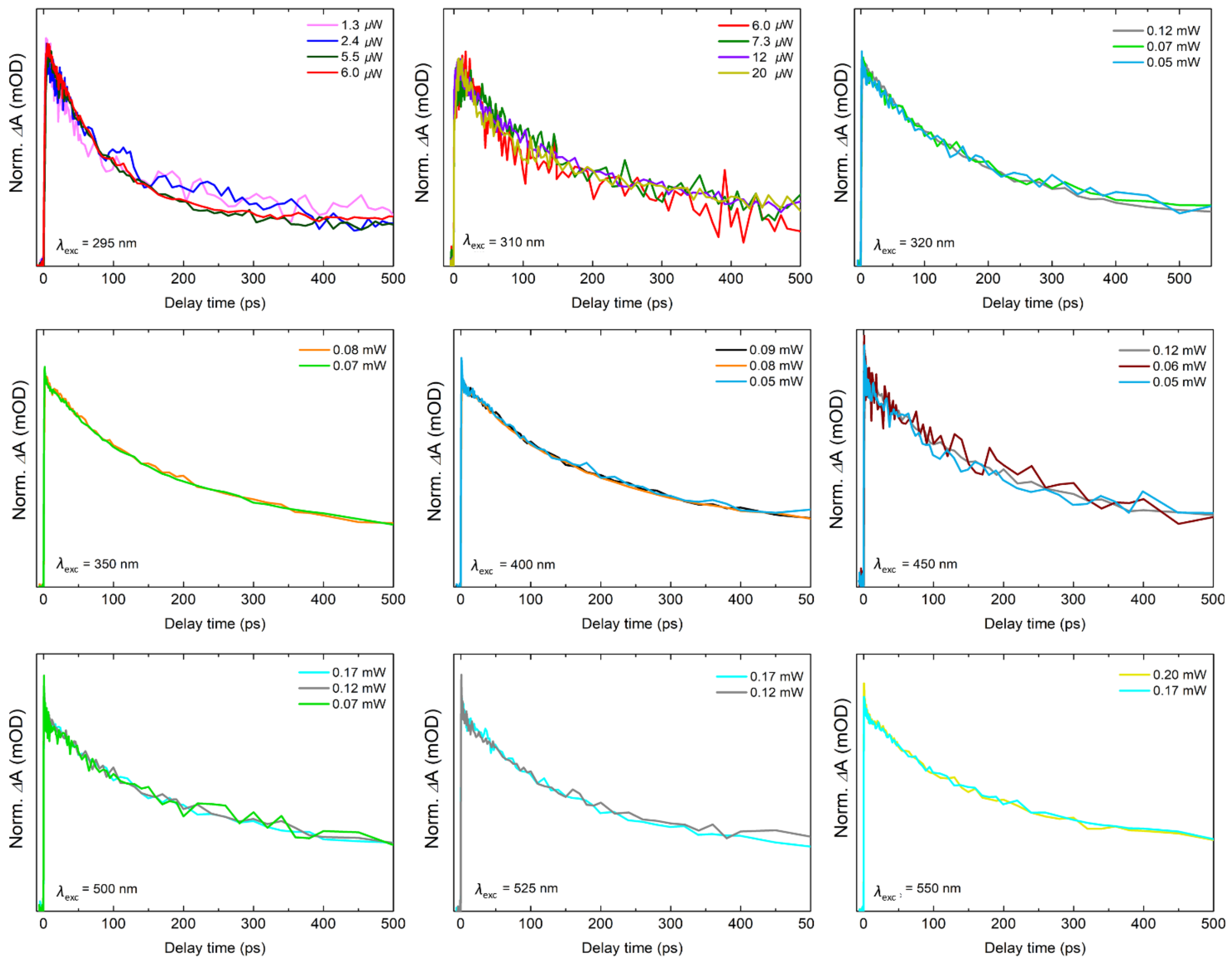

Supplementary Fig. 3: All photo-induced bleach transients. Normalized dynamics at the photo-induced bleach maximum, for each excitation wavelength in the linear regime. Here, we can clearly see the dynamics remain invariant under increasing pump fluence which confirms <*N*> << 1; e.g. the linear regime.

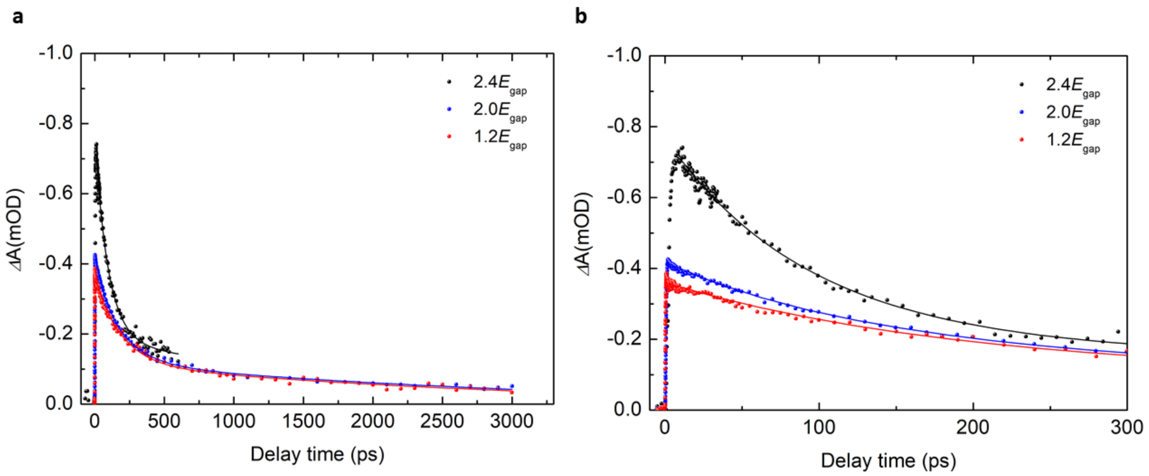

Supplementary Fig. 4: Photo-induced bleach dynamics normalized to fluence = 1. (a) The transient recorded at E_{pump} = 2.4 E_{gap} could be extrapolated and fitted using a triple exponential function, where the fast component corresponds to τ_1 = 89 ps and is the Auger recombination decay time. The slow components τ_2 = 189 ps and τ_3 = 3300 ps agree with the single exciton decay time which can be through non-radiative as well as radiative recombination processes. Similar values for slow decay time (189 ps and 3300 ps) have been found for excitation energies equal to and below the band gap energy. (b) The same transients as shown in (a) but on a shorter time scale.

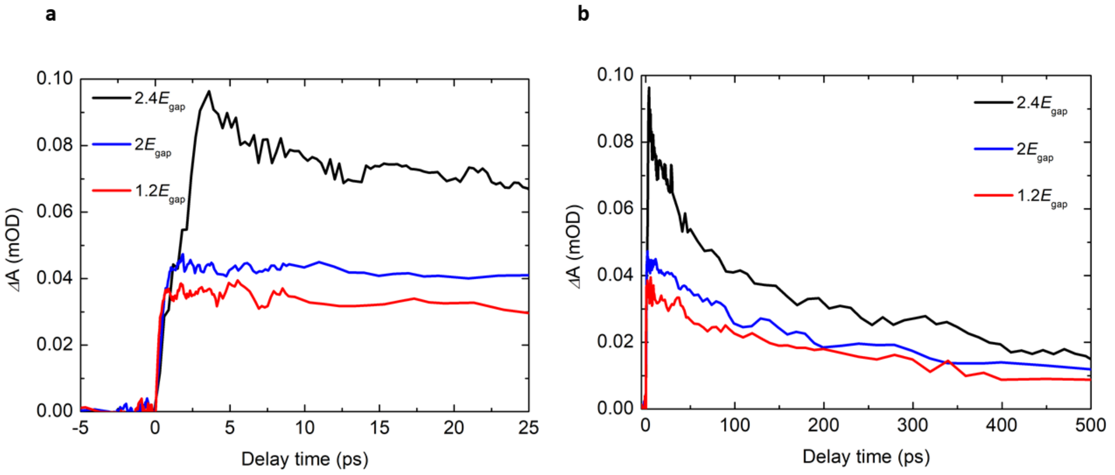

Supplementary Fig. 5: Transient photo-induced absorption dynamics. The dynamics at short (a) and maximum (b) delay time taken at the photo-induced absorption maximum (~620 nm, obtained by integrating the transients in the 610-630 nm range). Here, only the dynamics for above, at and below carrier multiplication threshold pumping are shown (because of the larger signal-to-noise ratio).

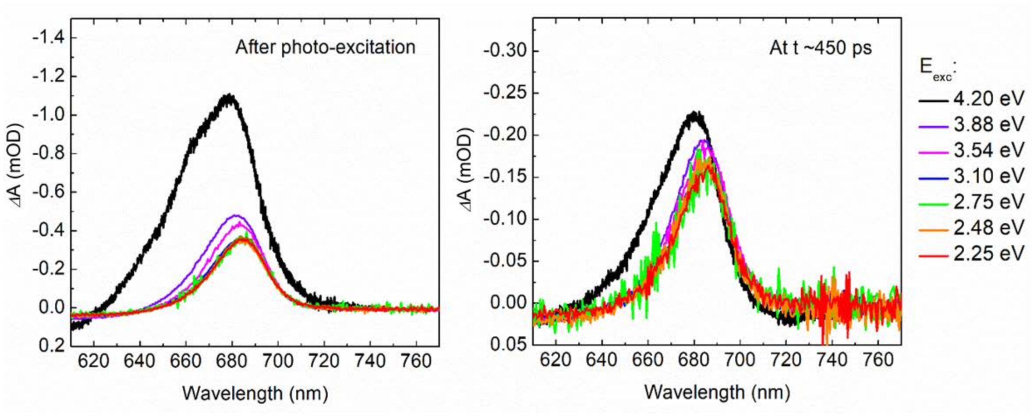

Supplementary Fig. 6: Transient absorption spectra. Spectra taken after the first few picoseconds i.e. almost directly after photo-excitation (left) and after t ~450, where the carrier multiplication and subsequent Auger recombination processes have completed (right). Clearly at early times, the photo-induced absorption (620 nm) is observed next to the photo-induced bleach (680 nm).

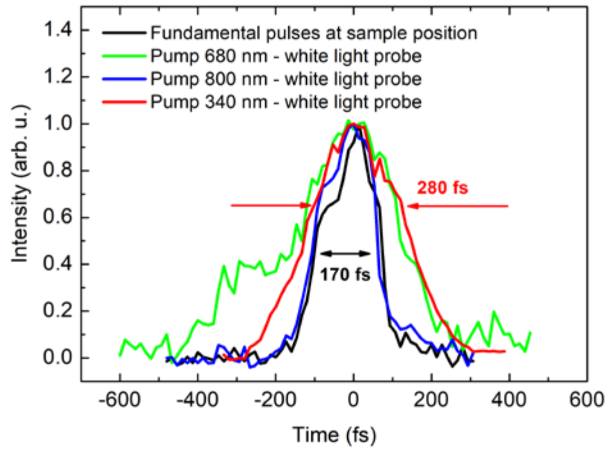

Supplementary Fig. 7: Determination of the setup responses. Cross-correlation of: (black) the fundamental pulses at the sample position (170 fs as taken from the full width at half maximum (FWHM, black); (green) pump at 680 nm and white-light probe (FWHM: 250 fs); (blue) pump at 800 nm and white-light probe (FWHM: 170 fs); (red) pump at 340 nm and white-light probe (FWHM: 280 fs). We also confirm that the pulse width at 350 nm is almost the same as at 340 nm (not shown here). Hence, a small difference between durations of pluses at 295-340 nm is expected. As such, the delayed rising of the photo-induced absorption is not due to an intrinsic nature of the material causing an elongated pulse width. The resolution of station 2 is described in ref.[1]

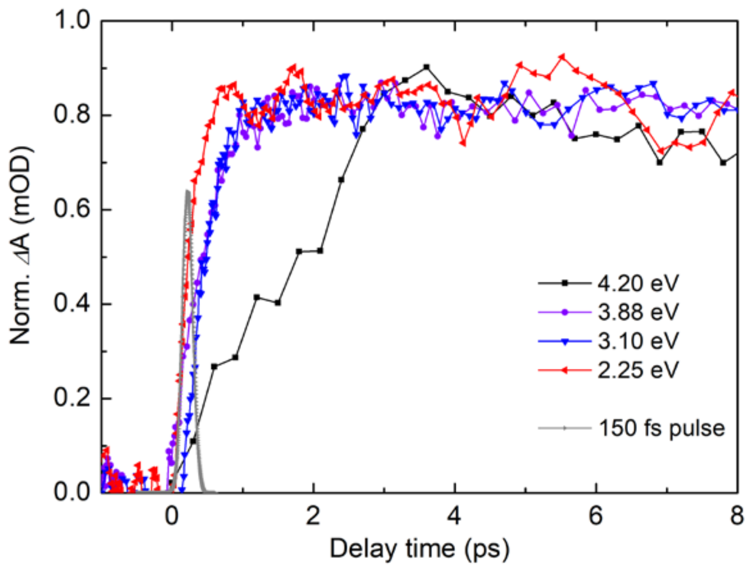

Supplementary Fig. 8: Initial rise of the photo-induced absorption signal. Similarly as for the photo-induced bleach a delay in the rise of the transient absorption signal when excited with higher energies is observed.

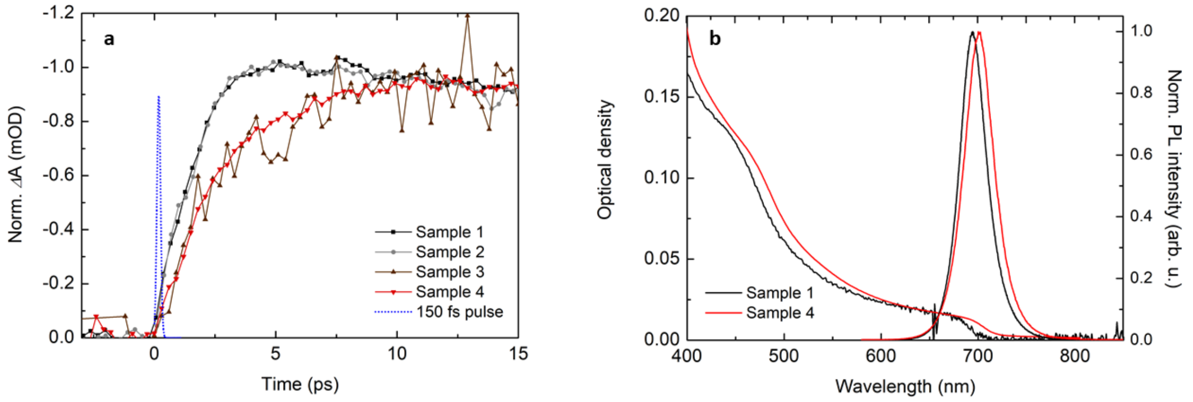

Supplementary Fig. 9: Comparison for different nanocrystal sizes. (a) Comparison of the photo-induced bleach rise times for different batches of nanocrystals prepared at different synthesis temperature: samples 1,2 (180 °C) and 3,4 (190 °C). We see that the results are consistent for similar prepared batches. The photo-induced bleach rise time of sample 1 (smaller nanocrystals) is faster than that of sample 4 (larger nanocrystals) which suggests that the carrier multiplication process speeds up as the nanocrystal size decreases. (b) Comparison of the photoluminescence (right) and absorption (left) spectra for samples 1 and 4.

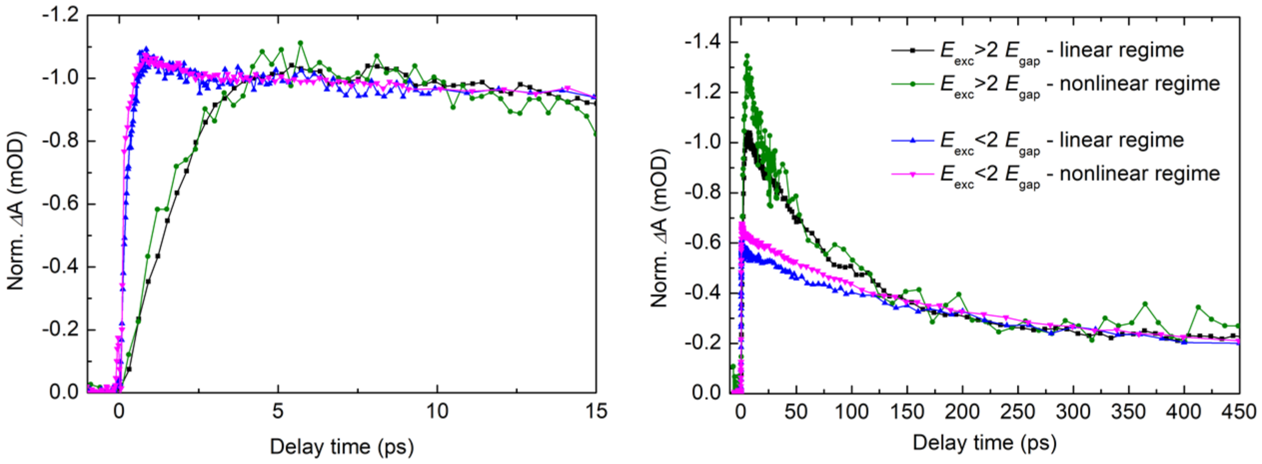

Supplementary Fig. 10: Build-up of the rise time. (Left) Comparison of the picosecond build-up of the photo-induced bleach rise time below (blue and pink transients) and above (black and green transients) the carrier multiplication threshold, while pumping in- and outside the linear regime. We see that the delayed rise time is observed only if carrier multiplication is taking place and that it is not induced through multi-photon absorption yielding multiple e-h pairs per nanocrystal. (Right) To show that we are really pumping outside the linear regime, the same transients are shown at a larger time scale and normalized to the tail.

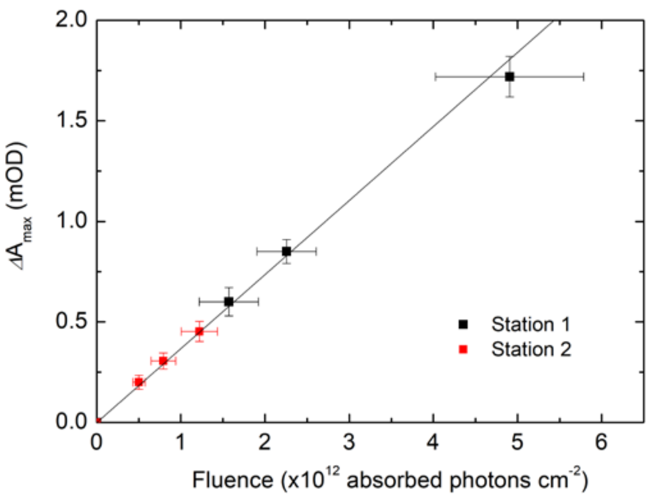

Supplementary Fig. 11: The number of generated carriers *vs* the absorbed photon fluence. At both stations, the number of generated carriers determined from the photo-induced bleach maximum as a function of the absorbed photon fluence is shown for 500 nm excitation. It can be seen that (even though not exact pump powers could be maintained) the data points follow the same linear dependence. The error bars in the y-direction are determined by the averaging between t = 4 - 8 ps after photo-excitation, to extract *ΔA*. In the

x-direction, the error is determined by the upper and lower values of the measured pump power in between consecutive measurements.

Supplementary references

1. Spoor, F.C., Tomić, S, Houtepen, A.J. & Siebbeles, L.D.A. Broadband cooling spectra of hot electrons and holes in PbSe quantum dots. *ACS Nano*, **11**, 6286-6294 (2017).
